# A Comprehensive Study on Radiological Hazard Assessment and Geological Features of Gypsum Deposits

**DOI:** 10.3390/toxics14030191

**Published:** 2026-02-25

**Authors:** Mohamed Y. Hanfi, Mohammad W. Marashdeh, Masoud S. Masoud, Hassan A. A. Shahin, Haitham Alrajhi, Ahmed E. Abdel Gawad

**Affiliations:** 1 Department of Life Safety, Institute of Fundamental Education, Ural Federal University, Ekaterinburg 620002, Russia; 2 Department of Physics, Dogus University, Dudullu-Ümraniye, Istanbul 34775, Türkiye; 3 Department of Physics, College of Sciences, Imam Mohammad Ibn Saud Islamic University (IMSIU), Riyadh 11623, Saudi Arabia; 4 Nuclear Materials Authority, El-Maadi, Cairo P.O. Box 530, Egypt

**Keywords:** gypsum deposits, radionuclides activity concentration, hazard assessment, environmental monitoring

## Abstract

This study assessed natural radioactivity values and corresponding radiological hazards in gypsum samples collected from the investigated area. The geologic context mainly includes tertiary and quaternary sedimentary formations with gypsum horizons of Early Messinian age, interbedded with layers of limestone and marl. A total of thirty-five gypsum samples were collected and analyzed for the ^238^U, ^232^Th, and ^40^K activity concentration using High-Purity Germanium (HPGe) gamma-ray spectrometry. The mean activity concentrations for the gypsums are reported at 73 ± 87 Bq kg^−1^, 14 ± 17 Bq kg^−1^, and 35 ± 201 Bq kg^−1^ for ^238^U, ^232^Th, and ^40^K, respectively. Several related radiological hazard indices were estimated from the various activity concentrations, including radium equivalent activity (Ra_eq_) and absorbed dose rate (D_air_). All gypsum analyzed fell below international safety limits for radiological risk, as evidenced by the observed radium equivalent activity (Raeq), with a maximum value of 456 Bq kg^−1^, and the total annual effective dose (AED) values from 0.09 to 1.26 mSv y^−1^ remaining between these two values. The results indicate the levels of radioactive hazards of the gypsum samples were generally below global safety standards, but individual samples (i.e., S17, S20, S24, S26, S30, S35) exceeded one or more of the hazard indices. Statistical assessment of the samples, with respect to their radiological hazard and natural radioactivity, was also undertaken as a way of seeking further insights into their relationships, productivity, and characteristics. This included Pearson correlation, hierarchical cluster analysis (HCA) and principal component analysis (PCA). The evidence suggests that for the gypsums, ^238^U was the greatest contributor to radiological hazards, influencing all hazard indices.

## 1. Introduction

Naturally occurring radioactive materials, or NORMs, contain radionuclides such as ^238^U, ^232^Th, and ^40^K that occur in varying concentrations in the Earth’s crust and are essential parts of rocks and minerals. They are widely distributed in mylonite, granite, pegmatite, rhyolite and basaltic volcanics, trachyte, soils, sandstone, marl, phosphate, and others, whereas uraninite, uranophane, autunite, thorite, uranothorite, zircon, and monazite are well present [1]. The main source of radiation that is not caused by humans comes from the environment, specifically, the Earth’s naturally occurring radioactive materials. However, there are also human-made radioactive materials (anthropogenic radionuclides) that were released into the environment due to previous nuclear testing and nuclear accidents [2,3,4,5].

Generally, the existence and natural radionuclide concentration show higher concentrations in igneous and metamorphic environs when compared with those of the sedimentary environs. The geochemical circumstances could influence the distributions of radionuclides during magmatic evolution and the hydrothermal alteration, as well as the chemistry of radioactive-bearing mineralization [6]. Human activities, including mining, agriculture, and industrial processes, have disturbed the natural equilibrium of radionuclides and redistributed them, increasing radiation in some areas. Anthropogenic factors in this case comprise any human activity that causes disruption of the natural balance between radionuclides, which contributes to an alteration or concentration of these radionuclides. Gypsum mining, quarrying operations, industrially processed raw gypsum, construction and development of infrastructure, use of phosphate fertilizer in agriculture, and utilization of gypsum-based material in both construction and soil conditioning are examples of anthropogenic factors. These activities may lead to a serious concentration of naturally occurring radioactive materials (NORMs) within our surroundings, thus resulting in higher levels of local radiation exposure [7,8,9,10].

The radiation dose exposure from natural sources accounts for the largest part of total dose radiation exposure in the general population. The majority of the exposure occurs from external sources (cosmic rays, terrestrial gamma radiation, etc.) and internal radionuclide (ingestion/inhalation) exposure to radionuclides. Radon gas is produced through the decay of uranium and can be naturally released from the earth and from building materials, entering the air surrounding these material sources; however, how much radon enters this air is affected by both the type(s) of materials used in construction and also the way in which the building is ventilated [11,12].

Monitoring and assessing radioactivity in environmental media, such as soil, water, air, and biological samples, is an important activity to identify the potential existence of an area with elevated radiation levels. These assessments can support the establishment of baseline data for radiological mapping, environmental impact assessments, and public health risk evaluations. These measurements can also provide valuable information regarding the natural background radiation levels, along with potential variations in space [13,14,15].

Natural radionuclides and their associated radiological hazards have gained popularity and relevance over the years, regarding growing awareness of the sustainability of our planet and the exposure of humans to radiation. Even low exposures of natural radiation can lead to a cumulative health effect of long-term exposure, particularly in areas with naturally high background levels or enhanced radioactivity from anthropogenic factors [16,17].

The sedimentary rocks such as limestone, sandstone, claystone, mudstone, shale, siltstone, conglomerate, phosphate, and white sand are considered natural resources for building materials, modern industries, and advanced applications such as bricks, cement, concrete products, gothic monuments, villa developments, schools, churches, tenement fronts, ceramic fabrication, painting, glassmaking, filtration, fertilizers, road construction, metal silicon, silicon weavers, oil and gas well drilling, water treatment, military, and electronic devices [18,19]. However, sandstone and phosphate rocks are considered the main resources that host U deposits such as roll-front sandstone and the Duwi and Abu Tartur phosphate deposits [20,21].

Gypsum (CaSO_4_·2H_2_O), a mineral produced by nature, was created by evaporation of seawater. The first known instance of its use was in the Roman Empire; now it has applications in construction, industry, agriculture, and medicine. The main uses include drywall, plaster, cement additives, pigments, and fillers to create pottery, artistry, and dental trays. In agriculture, gypsum is used to help with moisture retention in soil and prevent sodium buildup. Gypsum occurs as a sedimentary evaporite and has small traces of naturally occurring radionuclides associated with it; therefore, it is important in environmental radiological monitoring [22,23,24,25,26,27]. This study aims to analyze the activity concentrations of naturally occurring radionuclides in gypsum samples from different locations, while also examining the radiological hazard parameters. By employing HPGe spectrometry and standardized assessment procedures, this provides important data that support the safe use of gypsum as a building and industrial material, as well as enhancing environmental radiation monitoring and public health protection.

## 2. Geologic Setting

**Figure 1:**
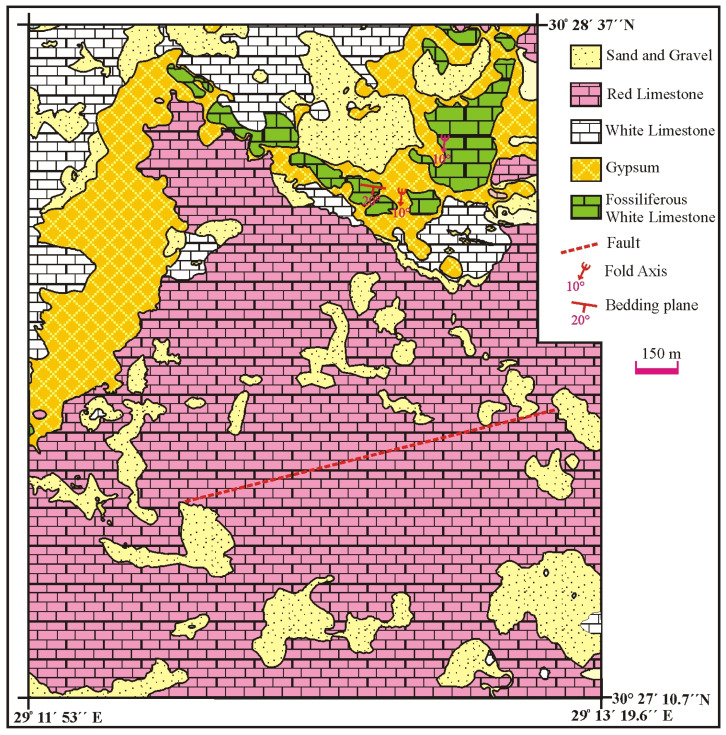
Detailed geologic map of Deir Al-Barkan quarries, NWD of Egypt.

**Figure 2:**
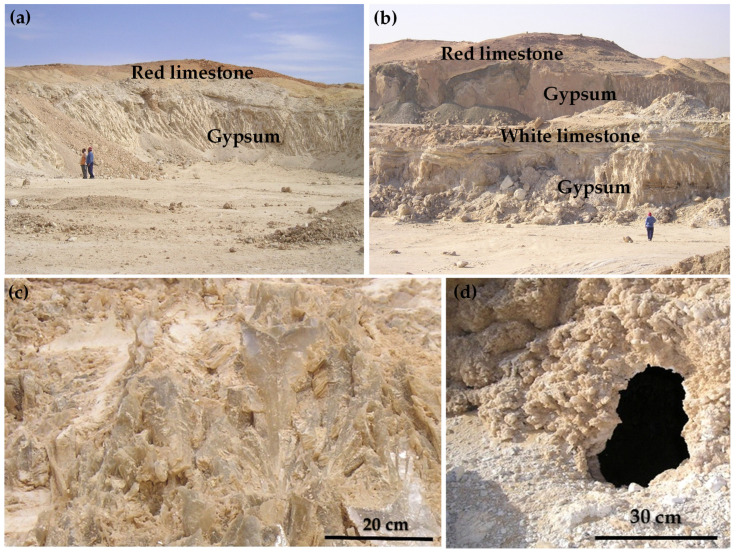
(**a**) Gypsum bed is overlain by red limestone; (**b**) two gypsum horizons interlayered with fossiliferous laminated limestone; (**c**) crystalline selenitic gypsum is very coarse, and overlies the evaporate sequence; (**d**) close-up view shows a remarkable cavity in the fine crystalline gypsum.

The investigated Deir Al-Barkan area is located southeast of El-Hammam town along the Mediterranean Sea coast at the Northwestern Desert (NWD) of Egypt (Figure 1). The Quaternary alluvial deposits, composed of aeolian sand and gravels, cover the Tertiary sedimentary rocks comprising red limestone, white oolitic limestone, clay, gypsum, fossiliferous limestone, marl, and sandstone. The northwest Mediterranean coastal zone is composed mainly of sedimentary rocks, especially silt, which span from the Tertiary to the Quaternary age [28]. In the study area, the Miocene deposits are overlain by Pleistocene oolitic limestone (Figure 1). The Quaternary deposits consist mainly of Pleistocene oolitic limestone, loamy deposits, and drift sands, which primarily cover the floors of narrow valleys and low-lying areas. This is followed by Miocene deposits of the Moghra Formation and Marmarica Formation. The base of this sequence comprises fluviatile to fluviomarine deposits that are representative of the Moghra Formation. It is composed mainly of sand, shale, and limestone, which are interbanded with plant and vertebrate fragments. The limestone is distinguished by a shallow marine origin, and its widespread areal expansion is a remarkable characteristic of the Marmarica Formation. It is composed of white limestone with lower calc-arenite of gray color with shale intercalation. In the Deir Al-Barkan area, the Marmarica Formation is overlain by two gypsum horizons, which are interlayered with oolitic limestone and sandy gypsiferous marl [29] (Figure 2a,b). The top of the evaporate sequence is an erosional surface unconformably overlain by stromatolitic carbonate, a pink conglomeratic limestone bed, and highly karstified pink limestone [30].

In the Deir Al-Barkan area, we are concerned with investigating the two gypsum horizons of the lower and upper gypsum beds. The gypsum deposits were formed during the Early Messinian time in a shallow marginal marine environment. The lower gypsum lens is snow white to pale gray, coarse crystalline, and sometimes graded into fine crystalline, showing a sugary texture (Figure 2c,d). The lower bed is stromatolitic gypsum and extends along the NE trend. Locally, this bed is intercalated with highly gypseous marl. This bed is overlain by white, undulated limestone beds. It is distinguished by remarkable fissures that are filled with gypseous sand and clay. Gypsum karst features are represented by vertical dissolution conduits of sub-parallel gypsum walls that range in width from 50 cm to 2.5 m and from 1.5 to 4 m. This karst is filled with clay, gypseous sands, and limestone detritus. The lower gypsum lens shows lateral variation in its thickness and varies from 2 m to reach up to 11 m in the northeastern part of the study area.

Upper gypsum lens comprises two main categories: skeletal-grass-like gypsum and coarse crystalline selenite-grass-like gypsum. The skeletal gypsum is light gray to white in color, with large crystals up to 6 cm and poorly developed crystal orientation. These crystals show selenite twins, which sometimes aggregate randomly, leaving intercrystalline spaces filled with fine gypsum crystals. The skeletal gypsum has a massive appearance and grades laterally and vertically to grass-like gypsum facies. The grass-like gypsum facies displays rows of coarse upright gypsum crystals. These rows are separated by fine crystalline gypsum layers up to 3 cm thick. The upright gypsum crystals are about 8 cm in height. These beds are gradually overlain by white to pale gray, bedded, and coarse to very coarse crystalline selenite gypsum and occur at the top of the evaporate sequence (Figure 2c). In parts, the massive selenite gypsum graded laterally into bedded grass-like gypsum, which was composed of rows of vertically oriented twins. These beds vary in thickness from 2 to 4 m. In some parts, the inter-crystal spaces are filled with carbonate and clay. Gypsum karsts are observed in the upper part of the gypsum quarry in the prospected area. It is well characterized by remarkable vertical karst and cavities. The vertical karst features are distinguished by vertical dissolution conduits of sub-parallel gypsum walls that range in width from 50 cm to 2 m and from 1.5 to 6 m. The open sub-vertical cracks and fissures are filled with gypseous sands, clays, and limestone detritus. Cavities are well observed in grass-like gypsum and display 30 cm in the greatest diameter. The wall of the cave was built up from white, fine- to medium-grained, sugary gypsum crystals, and botryoidal texture. The fine crystalline gypsum graded upward into coarse, grass-like gypsum. Most of the upper gypsum bed is overlain by clay, white limestone, red limestone, and Quaternary sediments. The vertical sequence of the prospected gypsum deposit may indicate a remarkable increase in salinity upward, brine restriction, and probable deepening of the evaporate basin [30].

Structurally, the upper gypsum bed in the prospective area is distinguished by a lenticular shape, ranging in form with maximum thickness in the middle part and thinning toward the peripheries. The gypsum lens has an E–W trend, and dipping ranges from 5° to 15° N. The red limestone, white limestone, clay and upper gypsum lens were significantly affected by joints and cracks having NE and N–S trends, with dipping angles ranging from nearly sub-vertical to 60° E. Some of these joints, cracks, and fractures were filled with clay and white limestone. Fault systems play a remarkable role and are responsible for low to high relief in the study area. Generally, the area is affected by NW and NE fault systems, and the intersection between both of them is filled with aeolian sand and gravel.

## 3. Materials and Methods

### 3.1. Sampling and Preparation

Samples of gypsum were obtained from various representative locations to ensure a wide distribution of geographical and material origin. In every location, about 1–2 kg of raw gypsum was obtained with clean, non-contaminating tools. The samples were stored in labeled, sealed polyethylene bags to avoid contamination or moisture from transport and storage. Upon arrival at the laboratory, the gypsum samples were first air-dried to ambient temperature for a period of several days to remove residual moisture. Upon drying, the specimens were crushed using a mechanical crusher, then ground to fine powder using an agate mortar and pestle to ensure particle size uniformity for better counting efficiency. The powdered specimens were then sieved using a 250 µm mesh to ensure that the sample was homogeneous. Drying proceeded with a known weight (generally ~500 g) of each homogenized sample being placed into a standard 500 mL Marinelli beaker. The samples were sealed and placed into storage for 28 days to allow radium and its decay products, namely ^226^Ra and ^214^Bi/^214^Pb in the uranium series, to achieve secular equilibrium.

### 3.2. Radioactive Measurements

Activity concentrations of naturally occurring radionuclides—^238^U, ^232^Th, and ^40^K—were based on previously established methods and directly measured in gypsum samples using High-Purity Germanium (HPGe) gamma-ray spectrometry (Canberra GC4018, Canberra Industries, Meriden, CT, USA). HPGe detectors are renowned for their excellent energy resolution, which facilitates identifying closely spaced gamma-ray peaks, in particular, the crucial aspect of potentially complex spectra common to natural materials such as gypsum. Before any measurement of the samples, the HPGe system was accurately calibrated for energy and efficiency using certified reference materials with standardized concentrations of radionuclides. The digital multichannel (MCA) analyzer and computer used to analyze the digital multichannel (MCA) spectrum are part of a system constructed using the Genie 2000 software. For each of the samples measured for 86,000 s (24 h), we used the previously mentioned industry standard counting time, which is generally accepted in gamma spectrometry for environmental measurements, to achieve acceptable counting statistics. A background spectrum was recorded for the same counting time under identical experimental conditions, and it was obtained using an empty sealed container. This background spectrum was subsequently removed from the sample spectrum to give net counts. The system of HPGe detectors had been calibrated before the sample measurements were made; this calibration included checks for energy calibration and efficiency.

The standard gamma-ray sources used for calibration were Cesium-137 (661 keV), Cobalt-60 (1173 keV and 1332 keV) and Europium-152 (121.8, 244.7, 344.3, 411.1, 443.9, 778.9, 867.4, 964.1, 1085.9, 1112.1, and 1408.0 keV), which had several peaks including the entire energy range. These standard isotopes will enable a reliable calibration of the entire energy spectrum of interest for environmental radioactivity measurements. To test and validate the accuracy and efficiency calibration of the complete measurement system, International Atomic Energy Agency (IAEA) reference materials were used: IAEA-RGU-1 (uranium), IAEA-RGTh-1 (thorium), and IAEA-K-1 (potassium). Activity concentrations of each radionuclide were calculated using the following equation [31]:

(1) $$A({Bq\;kg}^{-1})=\frac{C}{\varepsilon \cdot P\gamma \cdot m}$$

A is the activity concentration in Bq kg^−1^, C is the net count rate under the gamma peak (counts per second), ε is the detector efficiency at the gamma energy, Pγ is the probability of gamma emission for the radionuclide, and m is the sample mass in kg. This equation ultimately calculates radioactivity concentrations (in Bq kg^−1^) based on the gamma-ray spectrometric data provided.

The net peak area associated with each gamma line is determined by subtracting the portion of the peak area’s total that was attributable to background noise from the original value. In order to determine how background levels could differ from one detector to another, adjacent areas of the photopeak were measured for counts to provide a means of estimating the background. The net count is derived from the total measured counts minus the count attributed to background. Using the Full Width at Half Maximum (FWHM) method on standard peaks from the calibration sources, the energy resolution of the HPGe detector was determined. The FWHM values were approximately 1.33 keV at 122 keV (Cobalt-57), 2.0 keV at 661 keV (Cesium-137), and 2.5 keV at 1332 keV (Cobalt-60). The narrow FWHM values provide high confidence after discerning peaks appropriately. The relative efficiency of 40% at 1.33 MeV (^137^Cs), along with high energy resolution, provided not only accurate identification but also quantification of the radionuclides ^238^U, ^232^Th, and ^40^K in gypsum samples using an HPGe detector. This significantly adds to the ability to distinguish gamma emissions from ^238^U, ^232^Th, and ^40^K, even with interference from other complex features in the sample matrix. Quantification of radioisotopes was conducted by focusing on the characteristic gamma-ray energies that are emitted by their decay products. For example, ^238^U was quantified using the gamma lines of its own daughter nuclide ^214^Bi at 609 mesh energy peaks of 609 keV, 1120 keV, and 1764 keV. The ^232^Th series was quantified for connectivity orientation, using the high-energy lines from ^228^Ac at 583 keV and 911 keV, as well as the lines observed at 2614 keV from ^208^Tl. The activity concentration of ^40^K was assessed by its characteristic transverse gamma line at 1460 keV.

The Minimum Detectable Activity (MDA) represents the lowest radionuclide concentration that the HPGe detector can reliably measure. The MDA is calculated using the equation [31]:

(2) $$MDA=\frac{2.71+4.65\sqrt{B}}{\varepsilon \cdot P\gamma \cdot t\cdot m}$$

where t is the duration of time over which counts were recorded and ε is used to calculate m, Pγ, and B. Using the experimental settings utilized by the laboratory, MDA values for each radionuclide were determined based upon the type of radionuclides detected; for ^238^U and ^232^Th there were typical MDAs between 2 and 5 Bq kg^−1^ for radionuclides like ^238^U and ^232^Th, and around 5–10 Bq kg^−1^ for ^40^K, and these values will vary according to the energy of the gamma line detected and background levels for that specific gamma channel. These values provide an indication of the ability to detect specific radionuclides made during the analysis process. The uncertainties of the MDA values have been estimated with 95% confidence intervals as determined by statistical calculations based upon one standard deviation (±2σ), where σ is calculated as σ = $\sqrt{\frac{N_{S}}{T_{S}}+\frac{N_{b}}{T_{b}}}$ [31], with Ns and Ts representing the sample counts and measurement time, and Nb and Tb representing the background counts and measurement time, respectively [31].

### 3.3. Radioactive Hazards Assessment

Radium equivalent activity is a commonly adopted index, representing the radioactivity contributions of ^238^U, ^232^Th, and ^40^K in a single number (considering their associated radiological hazards) [32,33].

Ra_eq_ = A_U_ + 1.43 A_Th_ + 0.077 A_K_ (3)

where A_U_, A_Th_ and A_K_ are the respective activity concentrations (Bq kg^−1^) of ^238^U, ^232^Th, and ^40^K. The coefficients 1.43 and 0.077 represent the normalized hazard of thorium and potassium against uranium. The recommended safety limit set by the OECD for construction materials is 370 Bq kg^−1^ [5,34], meaning that values above this limit may result in an external dose rate exceeding 1 mSv y^−1^.

To determine the general radiological effect of naturally occurring radionuclides present in the sampled gypsum, a number of different radiological hazard indices have been calculated. All of these indices complement each other by presenting various means of determining an exposure level from direct gamma radiation, how much internal exposure occurs through radon inhalation, and how overall radiologically safe it may be to utilize as a construction material. The radium equivalent activity (Ra_eq_) serves as a comparative measure of the amount of specific activity (radiation produced) of the ^238^U, ^232^Th, and ^40^K radionuclides (with regard to the radiation hazards posed from gamma rays), while the absorbed dose rate (D_air_) serves to indicate how much gamma radiation the atmosphere (air) would receive from gypsum. The annual effective dose equivalent (AED) serves to indicate the amount of radiation an individual would receive as a result of using gypsum as a building material. The external hazard index (H_ex_) and internal hazard index (H_in_) serve to assess the potential risks associated with exposure to gamma radiation from gypsum and inhaling radon gas emitted from gypsum, respectively.

External hazard index (H_ex_): This index is utilized to keep the radiation hazard from external exposure to building materials within safe limits.

(4) $$H_{ex}=\frac{A_{U}}{185}+\frac{A_{Th}}{259}+\frac{A_{K}}{4810}$$

The value of H_ex_ should be ≤1 so that the external gamma dose stays below 1 mSv y^−1^.

The internal hazard index (H_in_) is the measure of internal exposure from inhalation of radon and its progeny, especially from ^238^U.

(5) $$H_{in}=\frac{A_{U}}{259}+\frac{A_{Th}}{237059}+\frac{A_{K}}{4810}$$

The limiting criterion is once again H_in_ ≤ 1

Gamma activity index (I_γ_): The European Commission recommends this index as a way to evaluate construction materials in a radiological protection context. Material can be deemed appropriate for construction purposes if I_γ_ ≤ 1. There may be limits on higher values depending on the application [35].

(6) $$I_{\gamma }=\frac{A_{U}}{300}+\frac{A_{Th}}{200}+\frac{A_{K}}{3000}$$

Absorbed dose rate in air (D_air_): This parameter is an estimate of the gamma dose rate in air at 1 m and above the ground surface due to the radionuclides in the material [36,37].

(7) $$D_{air}=0.430A_{U}+0.666A_{Th}+0.042A_{K}$$

This estimated dose rate value is an immediate measure of external exposure and is used to develop an annual effective dose.

Annual effective dose equivalent (AED): The AED is the annual dose from radiation received by a person, accounting for occupancy factors, and the conversion from absorbed dose to effective dose [36,37].

(8) $$AED\left(\frac{mSv}{y}\right)=\sum \left(D_{air}\left(\frac{nGy}{h}\right)\times 0.7\left(\frac{Sv}{Gy}\right)\times occupancy\;factor\right)\times 8760\;h\times 10^{-6}$$

where 8760 is the number of hours in a year, 0.2 and 0.8 are the occupancy factors for outdoors and indoors, and the conversion factor from absorbed to effective dose is 0.7 Sv/Gy. The recommended annual upper limit for AED from construction materials is 1 mSv y^−1^.

Annual gonadal dose equivalent (AGDE): AGDE indicates the yearly dose to reproductive organs, which is important when examining potential hereditary risks [38].

AGDE (mSv y^−1^) = 3.09 A_U_ + 4.18 A_Th_ + 0.314 A_K_ (9)

AGDE does not have a universal limit, but control values can be significant when falling above said value, as also indicative of chronic exposure, which likely has genetic and reproductive-related consequences.

Excess lifetime cancer risk (ELCR): ELCR predicts the chances of an individual developing cancer throughout their lifetime due to radiation exposure [39].

(10) $$ELCR\left({mSv\;y}^{-1}\right)={AED}_{out}\times DL\times RF$$

where DL = duration of life (assumed to be 70 years), RF = risk factor (0.05 Sv^−1^ as recommended by ICRP). This value is useful for estimating chronic exposure to long-term health risks.

## 4. Results and Discussion

### 4.1. Radioactive Content

**Figure 3:**
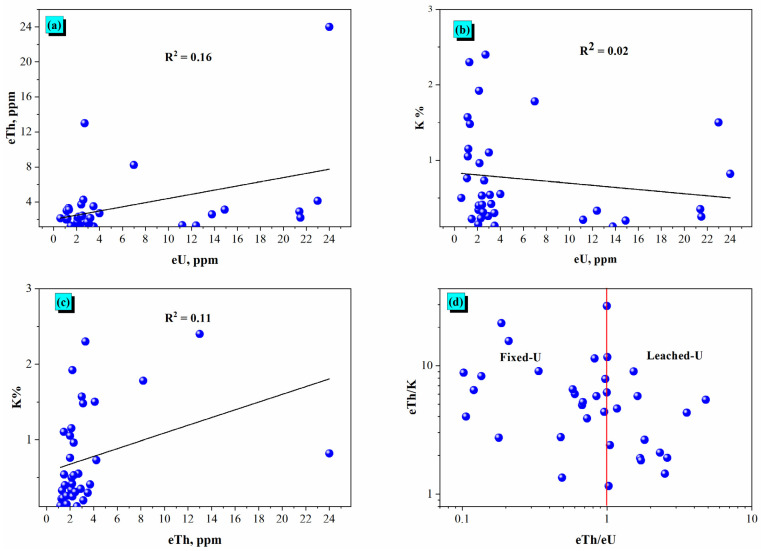
Correlations of radioactive content of (**a**) uranium content (eU, ppm), (**b**) thorium content (eTh, ppm) and (**c**) potassium content (K%), as well as (**d**) eTh/eU vs. eTh/K.

Figure 3a shows the relationship of equivalent Thorium (eTh) and equivalent Uranium (eU) concentrations in the gypsum samples. The R^2^ value of 0.16 means that a very weak positive correlation exists: variation in eTh can account for only a small amount of the variation in eU. The weak relationship suggested that geological processes that determine thorium and uranium distribution in these gypsum deposits are largely independent of one another or that the processes determining their relative abundance are highly influenced by other processes. In general, thorium is immobile and uranium is more mobile in oxidizing conditions; hence, their incorporation into the gypsum matrix and subsequent deportment were likely governed by different pathways.

The relationship between potassium content (K%) and equivalent uranium (eU) values is shown in Figure 3b. The extremely low R^2^ value of 0.02 indicates essentially no linear relationship exists between the two elements, implying that the amount of potassium in the gypsum samples is virtually independent of the uranium content. This result is consistent with the distinct geochemical characteristics of potassium, which is generally linked to silicate minerals, while uranium can exhibit mobility and associate with various other phases. The absence of a relationship suggests that sources and processes determining the incorporation of potassium and uranium into the gypsum are largely decoupled.

Figure 3c shows the relationship between potassium (K%) and equivalent thorium (eTh). It had an R^2^ of 0.11, which also indicated a very weak positive correlation, similarly to the eTh vs eU correlation shown previously. This suggests the concentrations of potassium and thorium in the gypsum samples are largely independent of one another, or their distributions are controlled by exogenous geology. The general immobility of thorium and potassium’s association with detrital minerals, however, would suggest this weak correlation also supports the notion that they are not co-located or co-transported in the gypsum environment, indicative of disparate geochemical pathways for incorporation.

Figure 3d further illustrates uranium mobility relating the eTh/K ratio with the eTh/eU ratio. The inclusion of the vertical red line at eTh/eU = 1 creates two zones at the eTh/eU border: the ‘Fixed-U’ zone (eTh/eU < 1), which indicates uranium that is referenced is relatively enriched or fixed, and the ‘Leached-U’ zone (eTh/eU > 1), indicating uranium is likely leached, or thorium is relatively enriched. Gypsum samples’ distribution, when interpreted in conjunction with the eTh/eU boundary, cannot be completely relied upon. The ratio of eTh/eU relative to eTh/K does not provide absolute values for any of the geomorphological processes that led to the deposition of these samples; however, it shows the qualitative range of changes in uranium mobility compared to thorium and potassium. Data points indicating eTh/eU> and <1 show that different factors may affect the distribution of uranium in gypsum, including local geomorphological conditions and mineralogical or post-depositional movement of the gypsum deposits. As such, the results from the eTh/eU and eTh/K plots must be combined with other methods, such as correlation, PCA, and hierarchical clustering analyses, for validation of interpretation and the relevance of these methods for identifying dependencies between different sedimentary components.

### 4.2. Radionuclide Concentrations and Hazard Assessment

Table 1 shows the activity concentrations of the natural radionuclides ^238^U, ^232^Th and ^40^K in the gypsum samples collected and analyzed (n = 35). The statistical analysis indicates considerable variation in radionuclide content in the samples, which may be attributed to the heterogeneous nature of the raw materials (e.g., gypsum) and several geological sources of raw materials. The gamma concentration value of ^238^U can be as low as 7 Bq kg^−1^ and as high as 296 Bq kg^−1^ (averaging 73 Bq kg^−1^ ± 87 Bq kg^−1^). The coefficient of variation (CV = 119%) implies a large amount of dispersion around the mean, indicating that uranium occurs heterogeneously in a gypsum matrix. The positive skewness (1.65) value implies a right-tailed distribution, which indicates a few samples with highly elevated concentrations relative to the bulk. The kurtosis value (1.38), close to a normal distribution (kurtosis ≈ 3), indicates the distribution is moderately peaked with limited extreme outlier points. In comparison to the global average of ^238^U in building materials of 35 Bq kg^−1^ [40,41], the mean activity concentration is above the global average; however, due to the range of the values from many samples having a large spread, there is a great deal of heterogeneity in the samples. As a result, the mean activity concentration can be interpreted with caution, and one cannot conclude that there is any one enrichment mechanism based on the large variability of these samples.

With an average concentration of 14 Bq kg^−1^, ^232^Th has a mean concentration that was the lowest out of the three radionuclides, with a range of 5–97 Bq kg^−1^ and a standard deviation of 17 Bq kg^−1^. It had a very high CV of 122%. There was pronounced variability in measurement, as the data have a strongly positively skewed distribution (skewness = 4.06), which suggests the data were influenced by some samples that had unusual thorium content. The high kurtosis value of 18.09 also supports a leptokurtic distribution with a sharp peak and heavy tails, indicating that there were outlier samples perhaps influenced by local inclusions of thorium-bearing minerals such as thorite monazite and zircon. The worldwide average value for ^232^Th in building materials is roughly 45 Bq kg^−1^ [40,42]. The mean in the current study is less than 50% of this average, meaning thorium is not a main contributor to the natural radioactivity of the gypsum samples. However, there are outliers, with values up to 97 Bq kg^−1^, suggesting caution in some uses. ^40^K has the highest mean activity of 235 Bq kg^−1^ (range: 38 to 751 Bq kg^−1^), and the highest standard deviation, 201 Bq kg^−1^. The CV (85%) is still high, but it is not as high as for ^238^U and ^232^Th. The positive skewness (1.22) indicates a moderately right-skewed distribution. While the kurtosis (0.54) indicates a flatter (platykurtic) distribution compared to the normal curve, it may imply a wider distribution with fewer outliers. The global average for ^40^K in building materials is 412 Bq kg^−1^ [40,43], and the average concentration of potassium in these gypsum samples is lower than this. These data indicate that potassium does not play a general radiological concern in this building material, even if some high concentrations would contribute to external or internal radiation exposure.

Among the three radionuclides, ^40^K is the most abundant in the gypsum samples, which is typical for potassium’s natural occurrence in the sedimentary material. The average value for ^238^U is also higher than for ^232^Th, which is expected in gypsum, perhaps due to geochemical mobility and uranium’s dissolved affinity towards conditions that form gypsum. The amount of variability in all three radionuclides, especially with the high CVs and skewness for ^232^Th, indicates that the raw gypsum has geochemically varied and may come from different mineral and sedimentary contexts. The elevated concentrations that were recorded in some of the samples have raised possible radiological concerns—especially in cases where the material is being used in bulk for construction or agricultural purposes. As a result, continuous monitoring and risk assessment are needed to ensure compliance with international safety standards relating to NORM.

The data within Table 1 demonstrates that the SD values are greater than the average measured activity concentrations for ^238^U and ^232^Th, indicating there is considerable variation within the spatial distribution of these radionuclides across the different gypsum samples being studied. Thus, the SD represents how dispersed the results are, while measurement uncertainty has been evaluated additionally for each of these radioisotope concentrations.

**Figure 4:**
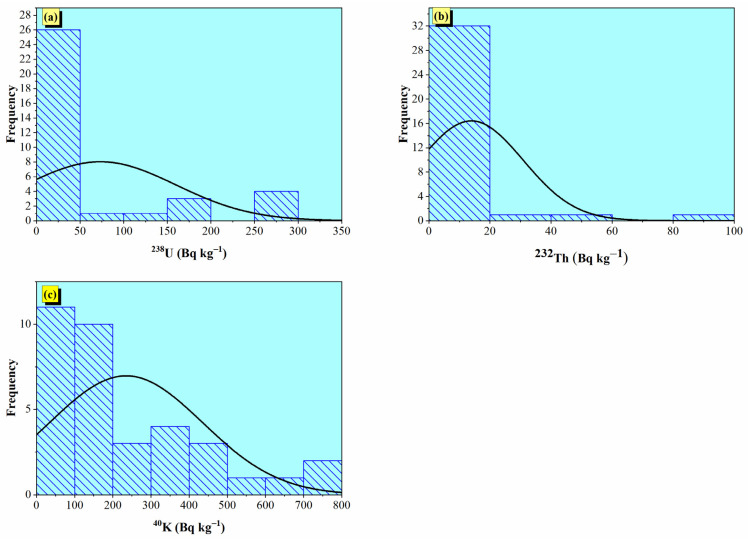
Frequency distribution of (**a**) uranium activity concentration (^238^U, Bq kg^−1^), (**b**) thorium activity concentration (^232^Th, Bq kg^−1^), and (**c**) potassium activity concentration (^40^K, Bq kg^−1^) in the gypsum samples.

Figure 4 shows that the frequency distribution of radionuclide activity concentrations in gypsum samples indicates that most samples had low quantities of ^238^U and ^232^Th. Most samples for ^238^U and ^232^Th clusters are below 50 Bq kg^−1^ and 20 Bq kg^−1^, respectively. This finding suggests that these elements have limited and relatively uniform natural radioactivity. Forty-Potassium has a broader and more variable distribution up to a maximum of 800 Bq kg^−1^, indicating that there may be greater heterogeneity in potassium content than in the other two elements. All three distributions are right skewed, which indicates that there is a limited number of samples with high radionuclide concentrations present (which may require assessments for radiological safety in construction applications).

**Figure 5:**
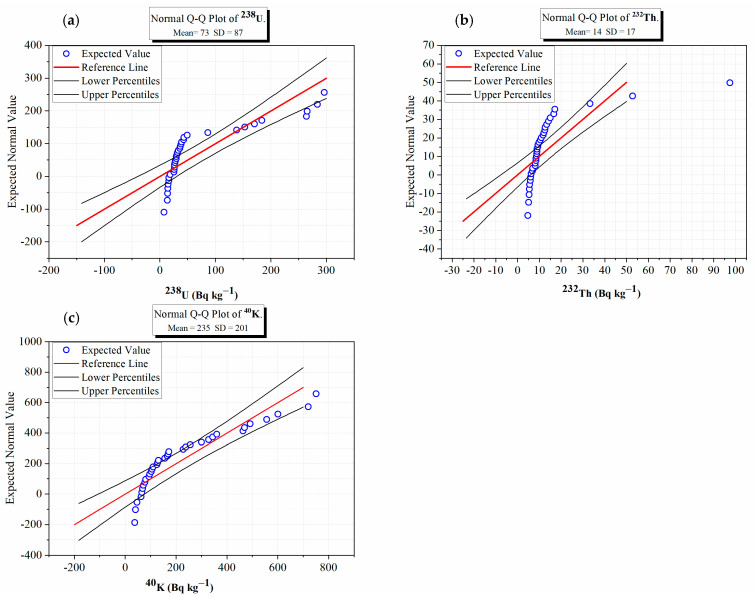
Q-Q plot of (**a**) uranium activity concentration (^238^U, Bq kg^−1^), (**b**) thorium activity concentration (^232^Th, Bq kg^−1^), and (**c**) potassium activity concentration (^40^K, Bq kg^−1^) in the gypsum samples.

The Q-Q plots for the activity concentrations of ^238^U, ^232^Th, and ^40^K (Figure 5a–c) aid in understanding the statistical distributions and the geochemical behaviors of these radionuclides. The plots of the data points for ^40^K demonstrate a close approximation to the theoretical normal distribution, with most of the data points falling within the lower and upper percentiles of the normal distribution, which indicates that the distribution of ^40^K is relatively homogenous because of the widespread availability of K-bearing minerals. The ^232^Th demonstrates significantly divergent behavior from the normal distribution, especially in low activity concentrations, indicating that ^232^Th lies along a heterogeneous distribution; this is likely due to the localized presence of accessory minerals that contain Th or due to variable sedimentary input. The ^238^U distribution is situated between the normal distribution and the ^232^Th distribution; the behavior of ^238^U shows moderate divergence from normality, indicating that U behaves with partial mobility (when it may have been transported) during its changing geochemical environment or post-depositional circumstances. The contrasting distributions illustrate that the three radionuclides exhibit differing geochemical controls that affect the distribution of U, Th, and K throughout the gypsum samples and further demonstrate how the Q-Q plots are an effective diagnostic method for determining whether distributions are heterogeneous. The outcome analyses were conducted using Q-Q plots shown in Figure 5a–c to assess normality of the activity concentration distributions for the radionuclides ^238^U, ^232^Th, and ^40^K and not to determine correlations between the radionuclides. The near vertical alignment of data points that are shown in a portion of the Q-Q plots, particularly for ^238^U and ^232^Th, reflects the fact that the majority of values are clustered at the lower end of the activity concentration distribution and that very few high activity concentration outliers were observed. These near-vertical trends represent deviations from a normal distribution and are supported by the high values of skewness and kurtosis that are presented in Table 1.

The Shapiro–Wilk (SW) test was used to statistically assess the normality of the radionuclide distributions in the gypsum samples, and the results are provided in Table 2. The SW test results revealed that all three radionuclides significantly contradicted a normal distribution, with *p*-values well below the standard significance level (α = 0.05). Specifically, ^232^Th has the smallest W statistic (0.47) and *p*-value (4.06 × 10^−10^), indicating a highly non-normal distribution, which is consistent to the evident right skew and high kurtosis. Likewise, ^238^U (W = 0.68, *p* = 1.72 × 10^−7^) reveals a statistically significant deviation from normality, therefore supporting the Q–Q plot and descriptive statistics. Although ^40^K results in the highest W statistic (0.84), its *p*-value (1.19 × 10^−4^) also indicates a significant deviation from normality, albeit this is the least concerning of the three radionuclides.

The present study compared the natural radioactivity levels in gypsum and among gypsum-based construction materials that have been measured in different parts of the world (in Table 3). The results of this study show that, with respect to gypsum and similar building materials, the activity concentrations of ^238^U, ^232^Th and ^40^K are within the expected ranges as reported in many other countries around the world. The average value of ^238^U (73 Bq kg^−1^) is higher than those reported from Iraq, Tanzania, and Pakistan, similar to values found in Nigeria and earlier studies conducted in Egypt, but lower than the highest values from India and Turkey. In contrast, the concentration of ^232^Th (14 Bq kg^−1^) is lower than most of the values previously reported, thus indicating a small contribution by thorium-containing minerals. The activity of ^40^K (235 Bq kg^−1^) is similar to the values reported for Iraq, Tanzania and Pakistan, though it is lower than the values from Turkey, India and Nigeria, where the potassium content is considerably greater. In general, uranium is found to be a dominant contributor to the radiological properties of gypsum studied, and while the contributions from thorium and potassium are less significant, these findings are consistent with those reported globally.

In order to evaluate the potential radiological risks of the gypsum samples, several common hazard indices and dose rate parameters were calculated and are presented in Table S1. These metrics can help to determine material suitability for building environments and any health implications.

Table S1 shows that the Ra_eq_ values in the samples varied from 31 to 456 Bq kg^−1^ with a mean of 111 Bq kg^−1^. This average is considerably below the intervention limit of 370 Bq kg^−1^ set by the [32,34,48] for building materials. However, some samples (S17, S20, S24, S26, S30, and S35) were above this limit and could pose higher radiological hazards due to their extensive utilization. This variation illustrates the heterogeneous distribution of radionuclides in the gypsum deposit, likely resulting from localized differences in mineralogy and/or alteration processes occurring after deposition.

**Figure 6:**
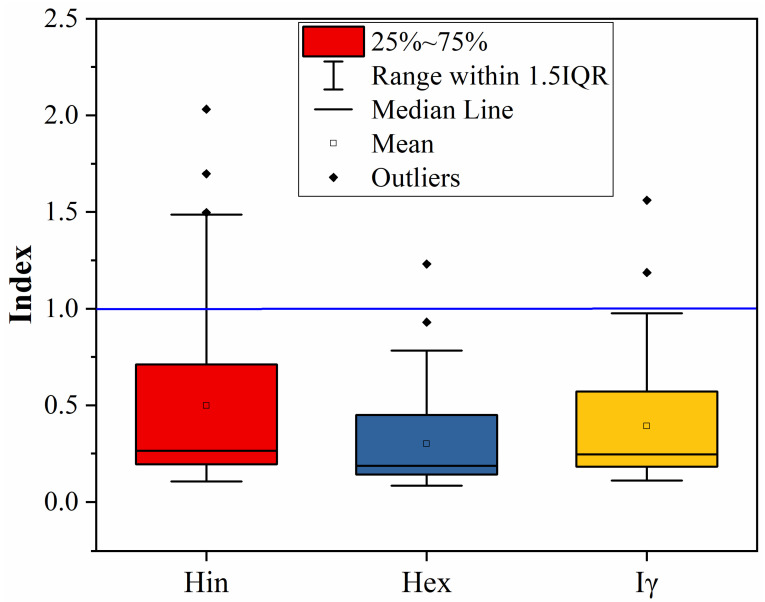
Hazard indices of gypsum samples at studied area. Bar plots show the mean value for each index with error bars representing the standard deviation, while box plots illustrate the spread and variability of the data. Blue line refers to the permissible limit of each index = 1.

Figure 6 reveals the internal hazard index (H_in_) ranged between 0.08 and 1.23 (mean = 0.30) while the external hazard index (H_ex_) ranged between 0.08 and 0.77 (mean = 0.50). The threshold level of both indices is 1, with levels above 1 indicating an increased probability of radiation hazards. The fact that S17, S20, S26 and S30 in Table S1 are near and/or exceed these thresholds raises concerns for potential localized radon daughter inhalation and/or gamma exposure, which could be hazardous in confined areas, such as homes.

The range of the I_γ_ values in Figure 6 was from 0.11 to 1.56, with a mean value of 0.39. EC guidelines state that I_γ_ > 1 values may need restricted use, especially in bulk considerations. The data in Table S1 indicate that while only a few samples (S17, S20, S26, and S30) fall into this category, most gypsum is considered safe for general use, and further scrutiny of samples will be needed prior to using them in building construction.

The air-absorbed dose rate, D_air_, in Table S1 varied between 14 and 206 nGy h^−1^, with an overall average of 52 nGy h^−1^. This average is modestly higher than the global average of 59 nGy h^−1^ for indoor exposure from building materials [5,49], but is still very reasonable overall for most samples. Nonetheless, these samples having exposure potential of 100 nGy h^−1^ or above (e.g., S17, S20, S24, S26, S30) have reasonable exposure potential.

Table S1 shows that the AED_out_ values varied, from 0.02 to 0.25 mSv y^−1^ (mean = 0.06), while AED_in_ values varied from 0.07 to 1.01 mSv y^−1^ (mean = 0.25). The higher doses indoors are attributed to occupancy factors. The recommended safety limit for public exposure is 1 mSv y^−1^ [5]. All three samples (S20, S17, and S30) are at or slightly beyond the acceptable limit inside buildings, indicating the potential for limited use or mitigation in the appropriate conditions.

The AGDE values in Table S1 range from 0.10 to 1.40 mSv y^−1^, with an average (mean) value of 0.36 mSv y^−1^. Higher AGDE values indicate a greater risk to more sensitive organs (reproductive tissue) and increased risk of harm to sensitive organs, including reproductive tissue. The AGDE values for samples S20, S17 and S30 are higher than 0.3 mSv y^−1^, as expected, which suggests that precautions regarding the use of these materials should also be taken into consideration when planning for the protection of sensitive tissues, including those related to reproduction.

ELCR values in Table S1 range from 0.06 × 10^−3^ to 0.895 × 10^−3^, with a mean of 0.22 × 10^−3^. The acceptable ELCR range, as per US EPA guidelines, is 10^−4^ to 10^−3^. The average value of the current work falls in the acceptable range and represents an overall acceptable risk. With regard to some samples (S20, S17, S26, and S30), they are closely approaching or exceeding this range, indicating careful material selection is necessary to ensure that exposure levels are kept within safety limits. Overall, the diverse distribution of natural radioactive substances within the gypsum sample group demonstrates this heterogeneity. While the majority of samples in this study were within international safety guidelines, some samples pose risks requiring cautionary measures, such as the need for careful consideration when using this material in construction or large-scale applications. This reinforces the necessity for site-by-site evaluations to be conducted prior to any decisions made on using gypsum material.

It is very important to emphasize that these vertical trends in the Q-Q plot do not necessarily indicate that there is a positive correlation between the radionuclide levels. Inter-element correlations (positive correlations amongst radionuclides) were assessed by performing independent Pearson correlation analyses (Table 4), and the statistical results clearly show that ^238^U has strong positive correlations with indicators of radiological hazard (Ra_eq_, H_in_, H_ex_, I_γ_, D_air_, AED, AGDE, ELCR); the r values range from as low as 0.93 to as high as 0.98 for hazard indices it is compared against, while ^232^Th has moderate positive correlations (r ≈ 0.64–0.66), and ^40^K has low or negligible positive correlations (r < 0.15) with these radiological hazard indicators, supporting the fact that its potential to contribute to radiation exposure overall from the gypsum samples was fairly limited. These results emphasize the much larger contributions of ^238^U, and then ^232^Th, towards the radiological safety of gypsum, and should be considered accordingly with regard to monitoring these radionuclides, especially based on the anticipated use of gypsum for indoor construction purposes.

Therefore, the Pearson matrix evaluates the relationships between natural radionuclides identified in gypsum samples. There is little to no association between the natural radionuclides ^238^U, ^232^Th and ^40^K, meaning these three radionuclides did not come from one sole source. The relatively low associations between uranium and thorium suggest the presence of separate mineralogical ores (or ‘hosts’) for each of the radionuclides and their differing geochemical behaviours (for example, uranium is more mobile during variable redox conditions while thorium is generally less mobile as it is incorporated into more stable sedimentary minerals). In addition to these points, the lack of association between both uranium and thorium within the gypsum sample indicates that potassium predominantly forms from different geological sources that are abundant within the global K-bearing mineral composition. Thus, this indicates that potassium from inert subsurface overburden will exist within the gypsum mineral matrix as an impeded phase rather than a mobile phase.

**Figure 7:**
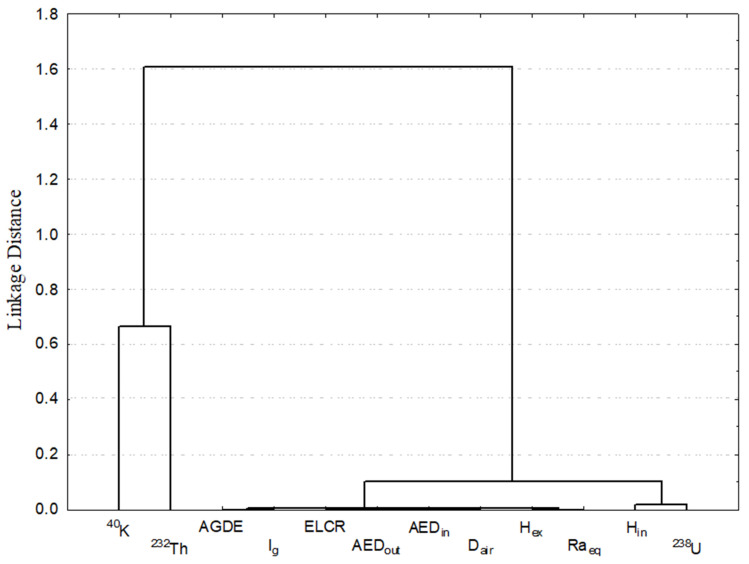
The clustering analysis of the radiological parameters for radiological data of gypsum samples at studied area.

The results of the hierarchical cluster analysis (HCA) and principal component analysis (PCA) assist in explaining the relationship between the various radionuclides and the various sample types. The HCA dendrogram in Figure 7 can be interpreted as grouping the samples according to similarities in radionuclide content and shows that there are two distinct clusters among the measured variables associated with gypsum samples. The first cluster includes ^40^K and ^232^Th, demonstrating similar behavior and presumably similar geochemical origins, with no direct radiological hazard index effects. The second cluster, with tighter connections between samples, included ^238^U and all the radiological risk parameters under consideration (D_air_, Ra_eq_, H_ex_, H_in_, AED, I_γ_, ELCR, AGDE), all of which are not only contrived but also highly correlated to uranium concentration. This means there is a significant relationship between the concentration of uranium and all radiological health risks. The results here show that the gypsum deposit was composed of different mineralogical constituents, with the possibility of variability in the depositional conditions of those constituents, resulting in compositional heterogeneity among the samples.

**Figure 8:**
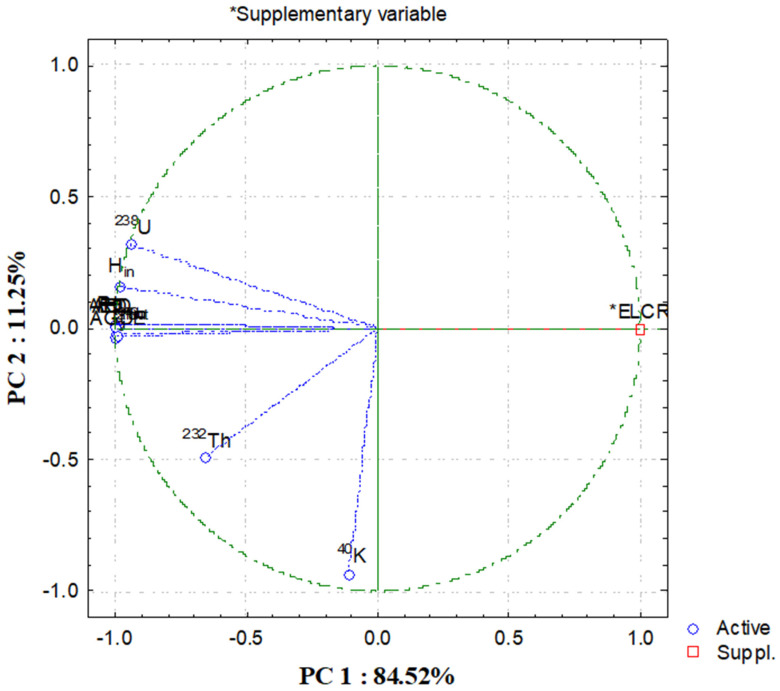
Principal component analysis (PC1 and PC2) for radiological data of gypsum samples at the studied area.

The PCA biplot in Figure 8 shows that the majority of the radiological hazard indices (including D_air_, AED_in_, AED_out_, H_ex_, H_in_, Ra_eq_, I_γ_, and AGDE) were positively correlated with ^238^U and clustered tightly along the primary axis (PC1), explaining a high amount of the variance (84.52%). This indicates the principal source of radiation-related risk in gypsum samples is from uranium. Conversely, ^40^K and ^232^Th exhibited low and independent behavior, located away from the primary cluster. The associated supplementary variable ELCR closely aligns with PC1, lending further evidence to the assertion that excess lifetime cancer risk is predominantly influenced by uranium concentration. In summary, this PCA confirms previous analyses and highlights ^238^U as being the main influencer of radiological safety of gypsum materials. Therefore, the combined results of the HCA and PCA provide evidence that the behavior of uranium was influenced by different distribution processes than those of thorium and potassium, thus allowing for greater understanding of the mechanisms governing the spatial distribution of these radionuclides and how these radionuclides were deposited into the gypsum deposit, in addition to the simple comparison of their concentrations.

## 5. Summary and Perspectives

This study includes an extensive radiological and statistical evaluation of natural radionuclides (^238^U, ^232^Th and ^40^K) contained within gypsum collected in the area of interest. It was found that ^238^U has much higher levels of activity concentration compared to ^232^Th and ^40^K, indicating that uranium plays the most significant role in defining the radiological characteristics of the gypsum. Whereas thorium had significantly lower levels of activity than that of uranium, potassium levels were moderate and typical for common K-bearing minerals. In addition to presenting activity concentration and radiological hazard index data, this work includes new findings based on a combination of statistical approaches, such as Q-Q plots, correlation analysis, hierarchical cluster analysis (HCA), and principal component analysis (PCA). These analyses clearly demonstrated differences between the types of distribution associated with each radionuclide, characterized by a near-normal distribution for potassium, an extremely heterogeneous distribution for thorium, and an intermediate level of variability for uranium. The differences among the radionuclides are indicative of the different types of mineralogical control exerted on the radionuclides and the varying levels of post-depositional mobility, thereby illustrating geochemical processes that are not readily apparent from the statistical summary alone and that previous studies have not adequately addressed. Based on radiological safety, the hazard indices (Ra_eq_, AED, H_ex_, H_in_, AGDE, and ELCR) calculated for the gypsum under review indicate the absence of any significant radiological hazard, and were consistent with the international recommended limits, although some samples (notably S17, S20, and S30) were above the limits, suggesting possible radiological risks if not properly screened. Based on these results, the gypsum is safe to use for construction and other related uses in its present condition. Additionally, this study represents an important baseline data set on the radiological safety of gypsum in the study area and provides valuable reference data for future environmental monitoring and regulatory evaluation activities. The findings from this investigation suggest that there are many opportunities for future research designed to reconstruct the historical trends in the natural radioactivity of the study area. Using stratigraphic or sedimentary records, the use of dating techniques, and supporting geochemical data as used in other environmental studies will allow for a more complete determination of the long-term natural variability of the radioactivity of the study site and the potential impacts of human activity on the distribution of radionuclides. By doing so, researchers will create an even greater understanding of the historical evolution of environmental radioactivity and support the development of sustainable resource utilization and environmental protection strategies.

## Figures and Tables

**Table 1:** Descriptive statistics of studied gypsum samples in the investigated area.

	N	Mean	SD	Min	Max	Skewness	Kurtosis	CV, %
^238^U (Bq kg^−1^)	35	73	87	7	296	1.65	1.38	119
^232^Th (Bq kg^−1^)	35	14	17	5	97	4.06	18.09	122
^40^K (Bq kg^−1^)	35	235	201	38	751	1.22	0.54	85

**Table 2:** Results of Shapiro–Wilk (SW) normality tests.

	DF	Statistic	*p*-Value
^238^U (Bq kg^−1^)	35	0.68	1.72 × 10^−7^
^232^Th (Bq kg^−1^)	35	0.47	4.06 × 10^−10^
^40^K (Bq kg^−1^)	35	0.84	1.19 × 10^−4^

**Table 3:** Comparison of activity concentrations (Bq kg^−1^) of natural radionuclides (^238^U, ^232^Th, and ^40^K) in gypsum and related building materials from the present study and selected studies worldwide.

No.	Country	^238^U	^232^Th	^40^K	Reference
		(Bq kg^−1^)	(Bq kg^−1^)	(Bq kg^−1^)	
1	Present study (Egypt)	73	14	235	This study
2	Iraq	19.7	20.1	232.2	[44]
3	Nigeria	47–63	24–32	219–257	[45]
4	Turkey	2.5–145.7	1.3–154.3	8.6–1044.1	[46]
5	India	13.8–151.9	14.2–207.7	55.3–1298.2	[47]
6	Tanzania	15–30	9–18	90–240	[26]
7	Pakistan	19–41	11–28	120–310	[25]
8	Egypt	58	21	312	[16]
9	Nigeria	28–63	25–59	300–760	[24]

**Table 4:** Pearson correlation between natural radionuclides and the radiological hazard coefficients of the gypsum in the studied area.

	^238^U	^232^Th	^40^K	Ra_eq_	H_in_	H_ex_	I_γ_	D_air_	AED_out_	AED_in_	AGDE	ELCR
^238^U	1.00	0.40	−0.15	0.95	0.98	0.95	0.93	0.95	0.95	0.95	0.94	0.95
^232^Th	//	1.00	0.34	0.65	0.54	0.65	0.66	0.64	0.64	0.64	0.65	0.64
^40^K	//	//	1.00	0.10	−0.02	0.10	0.15	0.12	0.12	0.12	0.15	0.12

## Data Availability

No data are associated with the manuscript.

## Supplementary Materials

The following supporting information can be downloaded at: https://www.mdpi.com/article/10.3390/toxics14030191/s1, Table S1: Radium Equivalent activity (Raeq) absorbed dose rate (Dair, nGy h^−1^), annual outdoor effective dose (AED in case outdoor and indoor, mSv y^−1^), and Excess lifetime cancer (ELCR) in the gypsum samples in the studied area.
