# Supplementary material for: A Comprehensive Study on Radiological Hazard Assessment and Geological Features of Gypsum Deposits
Source: Toxics. 2026 Feb 25;14(3):191. doi: 10.3390/toxics14030191 (PMC13030391; doi:10.3390/toxics14030191)
Supplement: Supplementary file 1 [file toxics-14-00191-s001.zip › toxics-4087613-supplementary.pdf]

Supplementary material

Table S1. Radium Equivalent activity (Ra_{eq}) absorbed dose rate ($D_{air,}$ nGy h^{-1}), annual outdoor effective dose (AED in case outdoor and indoor, mSv y^{-1}), and Excess lifetime cancer (ELCR) in the gypsum samples in the studied area.

Samples	Ra^{eq} Bq kg^{-1}	H_{in}	H_{ex}	I_{γ}	D_{air} (nGy h^{-1})	AED_{out} (mSv y^{-1})	AED_{in} (mSv y^{-1})	AGDE (mSv y^{-1})	ELCR
S1	53	0.26	0.14	0.18	25	0.03	0.12	0.17	0.11
S2	151	0.78	0.41	0.51	70	0.09	0.34	0.47	0.30
S3	44	0.15	0.12	0.17	21	0.03	0.10	0.15	0.09
S4	91	0.29	0.25	0.36	45	0.06	0.22	0.33	0.19
S5	31	0.13	0.08	0.11	14	0.02	0.07	0.10	0.06
S6	42	0.19	0.11	0.15	19	0.02	0.10	0.13	0.08
S7	169	0.87	0.46	0.57	78	0.10	0.38	0.53	0.34
S8	69	0.23	0.19	0.27	34	0.04	0.17	0.25	0.15
S9	78	0.35	0.21	0.28	37	0.04	0.18	0.25	0.16
S10	46	0.20	0.13	0.16	21	0.03	0.11	0.15	0.09
S11	72	0.29	0.19	0.27	35	0.04	0.17	0.25	0.15
S12	60	0.27	0.16	0.21	28	0.03	0.14	0.20	0.12
S13	45	0.19	0.12	0.16	21	0.03	0.10	0.15	0.09
S14	188	0.97	0.51	0.63	87	0.11	0.42	0.58	0.37
S15	52	0.24	0.14	0.18	24	0.03	0.12	0.17	0.10
S16	39	0.18	0.11	0.14	18	0.02	0.09	0.12	0.08
S17	290	1.50	0.78	0.98	134	0.16	0.66	0.90	0.57
S18	51	0.18	0.14	0.20	25	0.03	0.12	0.18	0.11
S19	55	0.19	0.15	0.21	27	0.03	0.13	0.20	0.12
S20	456	2.03	1.23	1.56	206	0.25	1.01	1.40	0.89
S21	86	0.30	0.23	0.33	42	0.05	0.21	0.31	0.18
S22	64	0.25	0.17	0.24	31	0.04	0.15	0.22	0.13
S23	62	0.28	0.17	0.22	29	0.04	0.14	0.20	0.12
S24	207	1.06	0.56	0.70	95	0.12	0.47	0.64	0.41
S25	32	0.11	0.09	0.12	15	0.02	0.07	0.11	0.06
S26	284	1.49	0.77	0.96	131	0.16	0.64	0.88	0.56
S27	56	0.23	0.15	0.20	26	0.03	0.13	0.18	0.11
S28	53	0.23	0.14	0.18	24	0.03	0.12	0.17	0.10
S29	70	0.23	0.19	0.27	34	0.04	0.17	0.25	0.15
S30	344	1.70	0.93	1.19	161	0.20	0.79	1.09	0.69
S31	71	0.31	0.19	0.25	32	0.04	0.16	0.22	0.14
S32	61	0.25	0.17	0.22	28	0.03	0.14	0.20	0.12
S33	74	0.29	0.20	0.27	35	0.04	0.17	0.24	0.15
S34	167	0.54	0.45	0.63	78	0.10	0.38	0.56	0.34
S35	177	0.71	0.48	0.64	83	0.10	0.41	0.58	0.36
Mean	111	0.50	0.30	0.39	52	0.06	0.25	0.36	0.22
SD	100	0.50	0.27	0.34	46	0.06	0.22	0.31	0.20
Min	31	0.11	0.08	0.11	14	0.02	0.07	0.10	0.06
Max	456	2.03	1.23	1.56	206	0.25	1.01	1.40	0.89
